# Predicting Drug-Target Interactions for New Drug Compounds Using a Weighted Nearest Neighbor Profile

**DOI:** 10.1371/journal.pone.0066952

**Published:** 2013-06-26

**Authors:** Twan van Laarhoven, Elena Marchiori

**Affiliations:** Institute for Computing and Information Sciences, Radboud University Nijmegen, Nijmegen, The Netherlands; Semmelweis University, Hungary

## Abstract

In silico discovery of interactions between drug compounds and target proteins is of core importance for improving the efficiency of the laborious and costly experimental determination of drug-target interaction. Drug-target interaction data are available for many classes of pharmaceutically useful target proteins including enzymes, ion channels, GPCRs and nuclear receptors. However, current drug-target interaction databases contain a small number of drug-target pairs which are experimentally validated interactions. In particular, for some drug compounds (or targets) there is no available interaction. This motivates the need for developing methods that predict interacting pairs with high accuracy also for these 'new' drug compounds (or targets). We show that a simple weighted nearest neighbor procedure is highly effective for this task. We integrate this procedure into a recent machine learning method for drug-target interaction we developed in previous work. Results of experiments indicate that the resulting method predicts true interactions with high accuracy also for new drug compounds and achieves results comparable or better than those of recent state-of-the-art algorithms. Software is publicly available at http://cs.ru.nl/~tvanlaarhoven/drugtarget2013/.

## Introduction

A core problem in pharmacology is the determination of interactions between drug compounds and target proteins in order to understand and study their effects. The in silico prediction of such interactions is of crucial importance for improving the efficiency of the laborious and costly experimental determination of drug-target interaction (see e.g. [1]–[4]).

Drug-target interaction data are available for various classes of pharmaceutically useful target proteins including enzymes, ion channels, GPCRs and nuclear receptors [5]. Publicly available databases have been built and maintained, such as KEGG BRITE [6], DrugBank [7], GLIDA [8], SuperTarget and Matador [9], BRENDA [10], and ChEMBL [11], containing drug-target interaction and other related sources of information, like chemical and genomic data.

The availability of these data has boosted the development of machine learning methods for the in silico prediction of drug-target interactions, including the seminal paper by Yamanishi et al. [12]. In that paper the authors distinguish between prediction for 'known' drug compounds or targets, for which at least one interaction is present in the training set; and prediction for 'new' drug compounds or targets, for which no interaction in the training set is available. This results in four possible settings for predicting drug-target interaction, depending on whether the drug compounds and/or targets are known or new.

The current state-of-the-art for the *in silico* prediction of drug-target interaction involves methods that employ similarity measures for drug compounds and for targets in the form of kernel functions, e.g., [12]–[19].

In this paper we generalize the applicability of the method introduced in [16] to so-called *new drug compounds*, that is, drug compounds for which no interactions are known. The method, hereafter called GIP, uses known interactions of a drug for predicting novel ones by means of a regularized least square algorithm incorporating a product of kernels constructed from drug compound and target interaction profiles. We propose a simple weighted nearest neighbor algorithm, called WNN, for constructing an interaction score profile for a new drug compound using chemical and interaction information about known compounds in the dataset. The WNN method can be used as a stand-alone algorithm for predicting interactions for new drug compounds. It can also be directly incorporated into the GIP method for handling new drug compounds. We call the resulting combination WNN-GIP. The methods can be directly adapted to handle also unknown targets or both unknown drug compounds and targets.

We test the predictive performance of WNN and WNN-GIP on four drug-target interaction networks in humans involving enzymes, ion channels, GPCRs and nuclear receptors. Results as measured by the area under the curve (AUC) and area under the precision-recall curve (AUPR) [20] show that the weighted nearest neighbor profile algorithm and its incorporation into the GIP method are capable to predict true interactions for new drug compounds with satisfactory accuracy. The algorithms achieve competitive or better results than the recent state-of-the-art algorithms KBMF2K [15] and BLM-NII [17]. KBMF2K is based on a fully probabilistic approach to model drug-target interaction, which can be applied to discover target (respectively drug compound) interactions for new drug compounds (respectively target proteins). Results in [15] indicate improved accuracy over the method introduced in [19]. BLM-NII is an extension of the BLM method [13] to deal with new drug compounds (or targets). In BLM-NII a drug-target interaction for a new drug compound is inferred by constructing an estimated interaction profile from the drug compounds in the training data. The resulting profile is then used as label information to learn an interaction model for that drug compound with the BLM method.

## Methods

### The Problem

We consider the problem of predicting interactions using a drug-target interaction network, chemical similarity between drug compounds and genomic similarity between targets proteins. Formally we are given a set 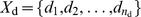 of drug compounds and a set 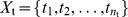 of target proteins. A set of interactions between drug compounds and targets is known. A bipartite network (between drug compounds and targets) can be constructed whose edges are such known interactions. Its corresponding adjacency matrix is a 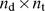 matrix 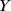 such that 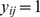 if drug compound 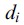 interacts with target 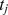, and 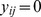 otherwise. Furthermore, information about the the chemical similarity between drug compounds and genomic similarity between targets is given in the form of the similarity matrices 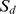 and 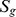, respectively.

The goal is to assign scores to drug-target pairs (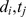) such that pairs with higher scores are more likely to interact.

### The GIP Method

Machine learning methods for tackling this problem are mainly based on the assumption that drug compounds exhibiting a similar pattern of interaction and non-interaction with the targets in a drug-target interaction network are likely to show similar interaction behavior with respect to new targets. A similar assumption on targets is considered. Here use the method introduced in [16]. It is based on the so-called (target) *interaction profile* 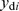 of a drug compound 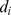, defined to be row 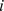 of the adjacency matrix 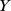, and the (drug compound) interaction profile 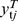 of a target protein 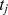, defined to be column 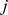 of 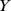. The interaction profiles generated from a drug-target interaction network are used as feature vectors for a classifier. A kernel from the interaction profiles is constructed using topology of the drug-target network, defined for drug compounds 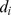 and 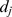 as follows:
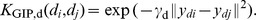
where



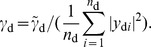



A kernel 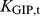 for the similarities between target proteins is defined analogously. Moreover, the kernels 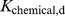 and 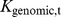 are considered, containing information about the chemical and genomic space. They are constructed from the chemical and genomic similarity matrices 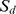 and 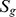 between drug compounds and between targets, by applying a simple transformation to make them symmetric and positive definite. The interaction profile kernel can be easily combined with these kernels using a weighted average.

The kernel for drug compounds and the kernel for target proteins can be combined using the Kronecker product 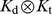, such that for drug-target pairs 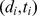 and 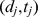


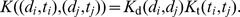



For each drug compound with at least one known interaction in the training data, a score interaction profile 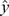 is computed from its interaction profile 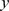 and the kernel matrix 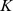, using the Regularized Least Squared (RLS) classifier. This is achieved by means of the simple closed form solution formula
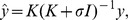
where 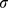 is a regularization parameter.

We refer the reader to [16] for a more detailed description and analysis of this method.

For simplicity in the sequel we call GIP the RLS algorithm that uses the kernel defined as the Kronecker product of the weighted averages of the interaction kernels and chemical and genomic kernels.

### Weighted Nearest Neighbor for New Drug Compounds

We want to extend GIP to new drug compounds, that is, compounds for which no interaction is known. To this aim, we propose a simple weighted nearest neighbor procedure. For a new drug compound, its chemical similarity with other known drug compounds and their corresponding profiles are used in order to infer a score interaction profile for that drug compound.

Specifically, the score interaction profile 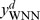 of a new drug compound 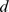 is the weighted sum of the profiles of the drug compounds in the training data, where a higher weight is assigned to profiles of those drug compounds more similar to 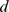. Let 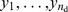 be the interaction profiles of the other compounds in the dataset (that is, the rows of 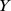), listed in decreasing order with respect to their chemical similarity to 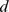. Then
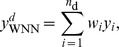
where the weights 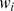
's are computed using a given decay value 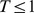 as 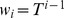. For computational reasons we only sum over drug compounds with weight at least 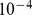. In our experiments we choose the decay rate 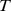 with 5 fold cross-validation to maximize AUC. We call the resulting procedure WNN.

An extension of GIP to handle new drug compounds using WNN, hereafter called WNN-GIP, can be directly formulated: for each new drug compound 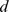, add 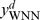 as new row to the matrix 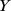 and apply GIP to predict the score interaction profile 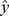 of 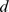.

### A Method to Show the Bias of a LOOCV Procedure

In a recent paper [17] the BLM-NII algorithm is introduced and assessed using the following leave-one-out cross validation (LOOCV) procedure. Each compound with only one interaction in 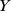 is treated as a 'new candidate' in the cross validation and the BLM-NII procedure is applied to it. We observe that in this way a strong prior is implicitly used in the cross validation, namely the fact that the considered compound had at least one interaction.

To illustrate how this prior introduces a bias on the results, we consider the following simple procedure, called Const. Const constructs an all '1's profile for the drug compounds or target proteins with only one interaction.

We can incorporate Const into GIP in the same way as WNN, giving the Const-GIP method. With this method all possible interactions for drug/targets with only one interaction will be ranked before interactions with drugs/targets that also have other interactions. Essentially, for such interactions the method only has to do half the work, since the fact that the drug/target is correct can be known with certainty. In real world situations there are also drug compounds that interact with none of the target under consideration, and vice versa, which would invalidate the Const-GIP method.

## Experiments

We perform a comparative experimental analysis of the proposed algorithms and two recently published methods [15], [17].

### Datasets

To this end we use the four drug-target interaction networks in humans involving enzymes, ion channels, G-protein-coupled receptors (GPCRs) and nuclear receptors from [12]. Table 1 lists some properties of the datasets.

**Table 1:** The number of drug compounds and target proteins, their ratio, and the number of interactions in the drug-target datasets from[12].

Dataset	Drugs	Targets	*n*_d_/*n*_t_	Interactions
Enzyme	445	664	0.67	2926
Ion Channel	210	204	1.03	1476
GPCR	223	95	2.35	635
Nuclear Receptor	54	26	2.08	90

Drug-target interaction information was retrieved from the KEGG BRITE [6], BRENDA [10], SuperTarget [9] and DrugBank [7] databases. Chemical structures of the compounds was derived from the DRUG and COMPOUND sections in the KEGG LIGAND database [6]. The chemical structure similarity between compounds was computed using SIMCOMP [21], which tries to find a graph matching between two compound structures. This resulted in a similarity matrix for the denoted by 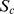, which represents the chemical space. Amino acid sequences of the target (human) proteins were obtained from the KEGG GENES database [6]. Sequence similarity between proteins was computed using a normalized version of Smith-Waterman score [22], resulting in a similarity matrix denoted 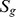, which represents the genomic space.

These datasets are publicly available at http://web.kuicr.kyoto-u.ac.jp/supp/yoshi/drugtarget/ and http://cbio.ensmp.fr/~yyamanishi/bipartitelocal/. They are used as current standard benchmark data for comparing the performance of machine learning algorithms for drug-target interaction. We use these datasets as they are without adding new interactions from source databases.

### Results

We follow the experimental procedure adopted in [15], [19]. Specifically, for each dataset, drug compounds are split into five subsets of roughly equal size. Each subset is then used in turn as the test set and training is performed on the data consisting of the remaining four subsets. This procedure is repeated five times.

Results are assessed using the AUC and AUPR quality measures, generally used in this type of studies. Specifically, the ROC curve of true positives as a function of false positives is computed, and the area under the ROC curve (AUC) is considered as quality measure (see for instance [23]). Furthermore, the precision-recall curve is computed, that is, the plot of the ratio of true positives among all positive predictions for each given recall rate. The area under this curve (AUPR) provides a quantitative assessment of how well, on average, predicted scores of true interactions are separated from predicted scores of true non-interactions. Since there are few true drug-target interactions, the AUPR is a more informative quality measure than the AUC, as it punishes much more the existence of false positive examples found among the top ranked prediction scores [20].

Average AUC and AUPR results and standard deviations are reported in Table 2. They indicate that a WNN-GIP has slightly better (average) AUC on all datasets except Enzyme. However, WNN has slightly better AUPR than WNN-GIP. By itself the GIP method does not work well in this setting, which is to be expected, since it was not designed to handle new drugs.

**Table 2:** Results of 5 fold cross validation: average AUC and AUPR over 5 runs.

Method	AUC (std)	AUPR (std)	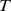 (std)
Enzyme			
GIP	0.685 (0.006)	0.150 (0.008)	
WNN	0.819 (0.004)	**0.299** (0.023)	0.809 (0.068)
WNN-GIP	**0.861** (0.004)	*0.280* (0.014)	0.908 (0.019)
KBMF2K	0.812 (0.004)	*0.287* (0.021)	
Ion Channel			
GIP	0.637 (0.008)	*0.179* (0.013)	
WNN	0.757 (0.006)	**0.249** (0.046)	0.535 (0.200)
WNN-GIP	0.775 (0.006)	*0.233* (0.024)	0.730 (0.171)
KBMF2K	**0.802** (0.006)	*0.245* (0.023)	
GPCR			
GIP	0.679 (0.014)	0.260 (0.023)	
WNN	0.848 (0.008)	0.308 (0.032)	0.713 (0.084)
WNN-GIP	**0.872** (0.008)	*0.311* (0.021)	0.702 (0.081)
KBMF2K	0.840 (0.009)	**0.347** (0.028)	
Nuclear Receptor			
GIP	0.758 (0.026)	0.357 (0.060)	
WNN	0.788 (0.027)	*0.434* (0.068)	0.305 (0.205)
WNN-GIP	**0.839** (0.023)	**0.456** (0.065)	0.527 (0.103)
KBMF2K	*0.810* (0.025)	0.354 (0.063)	

Standard deviation is reported between parentheses. The best AUC and AUPR results are indicated in bold, results that are not significantly different from the best (at 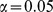) are indicated in italic.

To estimate the statistical significance of the AUC results we used the method described in [24]. To determine significance of the AUPR results we used bootstrapping.

The last column of table 2 lists the average value of the decay rate 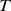 over the folds and repetitions. In general, the larger dataset have a higher (slower) decay rate, which means that more neighbors are taken into account.

### Comparison with other Methods

We consider the two following recent methods: KBMF2K [15] and BLM-NII [17].

KBMF2K is based on a Bayesian formulation that combines dimensionality reduction, matrix factorization and binary classification for predicting drug-target interaction networks using only chemical similarity between drug compounds and genomic similarity between target proteins.

In BLM-NII a drug-target interaction for a new drug compound 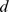 is inferred by constructing an estimated interaction profile for 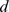 as follows. For each target, an entry of the profile for 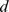 is defined as the sum of the similarity values of 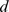 and each of the drug compounds interacting with that target. The resulting profile is then used as label information to learn an interaction model for 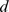 by means of the BLM method.

#### Comparison with KBMF2K

To compare results of WNN and WNN-GIP with those reported in [15], we follow the experimental procedure therein used (described in the previous section). Table 2 also includes the AUC and AUPR for the KBMF2K method. They indicate similar performance of KBMF2K and the simpler WNN algorithm, and slightly better overall results achieved by WNN-GIP, except on the Ion Channel dataset.

We could test the prediction capability of the proposed methods on unknown drug-target interactions of the given network using the procedure adopted in [15]. Therein, the complete interaction network for each dataset is used as training data, and the predictions on non-interacting pairs in the training set are ranked with respect to their interaction scores. However, since each drug compound or target in the training set has at least one interaction, we do not need to use WNN and the results are those of GIP. We report the top five predicted interactions for each dataset in Table 3. The full lists of all predicted interactions ranked by interaction score can be found in http://cs.ru.nl/~tvanlaarhoven/drugtarget2013/.

**Table 3:** Highest ranked predicted new interactions for each of the datasets.

	Rank	Drug compound		Target protein	
Enzyme					
M	*1*	*D00574*	*Aminoglutethimide*	*hsa1589*	*cytochrome P450, family 21, subfamily A, polypeptide 2*
C,M,D	*2*	*D00542*	*Halothane*	*hsa1571*	*cytochrome P450, family 2, subfamily E, polypeptide 1*
M,D	*3*	*D00139*	*Methoxsalen*	*hsa1543*	*cytochrome P450, family 1, subfamily A, polypeptide 1*
M	*4*	*D00437*	*Nifedipine*	*hsa1585*	*cytochrome P450, family 11, subfamily B, polypeptide 2*
C,M,D	*5*	*D00437*	*Nifedipine*	*hsa1559*	*cytochrome P450, family 2, subfamily C, polypeptide 9*
Ion Channel					
D,K	*1*	*D00438*	*Nimodipine*	*hsa779*	*calcium channel, voltage-dependent, L type, alpha 1S subunit*
	2	D00726	Metoclopramide	hsa1138	cholinergic receptor, nicotinic, alpha 5 (neuronal)
C,D	*3*	*D03365*	*Nicotine*	*hsa1137*	*cholinergic receptor, nicotinic, alpha 4 (neuronal)*
	4	D02098	Proparacaine hydrochloride	hsa8645	KCNK5: potassium channel, subfamily K, member 5
K	*5*	*D00552*	*Benzocaine*	*hsa6331*	*sodium channel, voltage-gated, type V, alpha subunit*
GPCR					
C,M,D	*1*	*D00283*	*Clozapine*	*hsa1814*	*dopamine receptor D3*
C,D	*2*	*D02358*	*Metoprolol*	*hsa154*	*adrenoceptor beta 2, surface*
	3	D00604	Clonidine hydrochloride	hsa147	adrenoceptor alpha 1B
C	*4*	*D03966*	*Eglumetad*	*hsa2914*	*glutamate receptor, metabotropic 4*
C	*5*	*D00255*	*Carvedilol*	*hsa152*	*adrenoceptor alpha 2C*
Nuclear Receptor					
	1	D00316	Etretinate	hsa6096	RAR-related orphan receptor B
C	*2*	*D00182*	*Norethindrone*	*hsa2099*	*estrogen receptor 1*
K	*3*	*D00348*	*Isotretinoin*	*hsa5915*	*retinoic acid receptor, beta*
	4	D01132	Tazarotene	hsa6097	RAR-related orphan receptor C
K	*5*	*D00348*	*Isotretinoin*	*hsa5916*	*retinoic acid receptor, gamma*

Interactions found in ChEMBL, Matador, DrugBank and KEGG are indicated in italic and marked as C, M, D and K respectively.

#### Comparison with BLM-NII

Table 4 shows the results of the LOOCV experiments. As expected, both Const-GIP and BLM-NII achieve very good results, with comparable AUC, and slightly better AUPR performance achieved by Const-GIP. To asses the statistical significance of these differences we used an upper bound on the variance of the AUC and AUPR for BLM-NII, because the actual variance is unknown. With this bound the differences in AUC scores are not statistically significant.

**Table 4:** Results of LOOCV on pairs.

Method	AUC	AUPR
Enzyme		
GIP	0.978	0.915
WNN	0.558	0.141
WNN-GIP	0.983	0.944
Const	0.577	0.179
Const-GIP	**0.991**	**0.969**
BLM-NII	*0.988*	0.929
Ion Channel		
GIP	0.984	0.943
WNN	0.528	0.125
WNN-GIP	0.986	0.953
Const	0.535	0.138
Const-GIP	**0.991**	**0.966**
BLM-NII	*0.990*	0.950
GPCR		
GIP	0.954	0.790
WNN	0.580	0.219
WNN-GIP	0.972	0.863
Const	0.604	0.266
Const-GIP	**0.988**	**0.910**
BLM-NII	*0.984*	0.865
Nuclear Receptor		
GIP	0.922	0.684
WNN	0.694	0.478
WNN-GIP	0.958	0.857
Const	0.744	0.568
Const-GIP	**0.989**	**0.926**
BLM-NII	*0.981*	0.866

Results of BLM-NNII are from [17]. The best AUC and AUPR results are indicated in bold, results that are not significantly different from the best (at 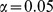) are indicated in italic, see the main text for details.

In general, these results indicate that cross validation should be applied and interpreted with care. Note that the cross validation procedure used in the comparison with KBMF2K is also positively biased, since we know that each 'new' drug compound has at least one interaction, but there the bias is much smaller.

## Discussion

In this work, we proposed a simple yet effective procedure to predict interaction profiles for unknown drug compounds and show how it can be directly integrated into a recent machine learning algorithm for the in-silico prediction of drug-target interactions. The novelty of our approach comes in the use of a weighted nearest neighbor procedure for inferring a profile for a drug compound by using interaction profiles of the compounds in the training data, where each profile is weighted using information about chemical similarity between drug compounds integrated with a simple decay scheme. The method can be directly modified to predict interaction scores of unknown targets (or of both unknown targets and drug compounds).

We performed a comparative assessment of the proposed methods on four different drug-target interaction networks from humans involving enzymes, ion channels, GPCRs and nuclear receptors. Results indicated that WNN is competitive in predicting interaction for unknown drug compounds with more involved machine learning methods recently proposed, notably a fully probabilistic method based on a Bayesian formulation that combines kernel-based nonlinear dimensionality reduction, matrix factorization and binary classification. Furthermore we showed that the direct integration of WNN in a recent kernel based machine learning method provides a general and powerful tool for finding drug-target interactions.

The computational complexity of WNN is 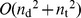, while the computational complexity of WNN-GIP is dominated by the RLS prediction using the Kronecker product kernel, which is 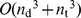 as implemented in [16], but can be further improved yielding a quadratic computational complexity by applying recent techniques for large-scale kernel methods for computing the two kernel decompositions, e.g. [25]. Therefore WNN-GIP is more efficient than KBMF2K, since the total time complexity of *each iteration* in the variational approximation method used in KBMF2K is 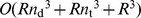, where 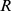 is the subspace dimensionality used in the method.

A limitation of our approach is that it does not make a difference between an inactive target and a target that has not been measured for a compound.

Compounds with a higher mutual chemical similarity also have a higher chance of having the same bioactivity. This information could be considered by WNN by determining directly the weights from the similarity, instead of using the proposed ranking-based decay mechanism. In this way all the compounds with high similarity would be considered with a high weight and all the compounds with low similarity would only have a minor contribution to the final predicted profile. On the same reasoning there is also a similarity threshold from where the chance is so low that two compounds have the same profile that it would be better not to predict something in the first place. In particular for new screening data from very large screening libraries chances are high that none of the references are really similar to the screening hits, which would most likely have a detrimental effect in the overall prediction performance, if predictions would be made for all such compounds. Many published target prediction algorithms apply such "applicability domain" or confidence estimations for their predictions. WNN could be modified to address this issue for instance by including a binary annotation based on a similarity threshold, or a more advanced procedure based on the similarities of all compounds considered for the generation of the profile.
